# Trial Evaluation of Protection and Immunogenicity of Piscine Bivalent Streptococcal Vaccine: From the Lab to the Farms

**DOI:** 10.3390/vaccines10101625

**Published:** 2022-09-28

**Authors:** Korntip Kannika, Soranut Sirisuay, Hidehiro Kondo, Ikuo Hirono, Nontawith Areechon, Sasimanas Unajak

**Affiliations:** 1 Department of Aquaculture, Faculty of Fisheries, Kasetsart University, 50 NgamWong Wan Road, Lat Yao, Chatuchak, Bangkok 10900, Thailand; 2 Graduate School of Marine Science and Technology, Tokyo University of Marine Science and Technology, Konan 4-5-7, Minato-Ku, Tokyo 108-8477, Japan; 3 Department of Biochemistry, Faculty of Science, Kasetsart University, 50 Ngam Wong Wan Road, Lat Yao, Chatuchak, Bangkok 10900, Thailand; 4 Kasetsart Vaccines and Bio-Product Innovation Centre, Kasetsart University, 50 Ngam Wong Wan Road, Chatuchak, Bangkok 10900, Thailand

**Keywords:** Nile tilapia, streptococcosis, bivalent vaccine, immune response, field trial

## Abstract

Streptococcosis is one of the major diseases that causes devastation to farmed fish, leading to significant economic losses all around the world. Currently, two serotypes of *Streptococcus agalactiae*, serotype Ia and III, have been identified as virulent strains and major causative agents of the disease in farmed Nile tilapia (*Oreochromis niloticus* Linn.) in Thailand. Upon inactivated vaccine development, monovalent inactivated whole-cell vaccines demonstrated high specific antibody production against homologous serotypes and limited production with heterologous serotypes. However, for higher efficacy, a bivalent streptococcal vaccine was designed to maximize protective immunity to both serotypes. Interestingly, our bivalent vaccine could successfully induce specific antibody production against both serotypes with similar levels, and the response could extend over the 8 weeks of the experimental period. Evaluation of vaccines in the laboratory scale revealed relative percent survival (RPS) of vaccinated tilapia to serotype Ia (81.2 ± 9.4%) and serotype III (72.2 ± 4.8%), respectively. The efficacy of the bivalent vaccine showed significant RPS higher than the monovalent vaccine (*p* < 0.05) at 30 days, and the protection of all those vaccines was reduced thereafter. Evaluation of the vaccine in a farm trial in different locations in Thailand revealed the efficacy of the bivalent vaccine in increasing the production yield by greater than 80% in all tested farms in 2015 and 2021. Taken together, this study affirms the efficacy of the bivalent streptococcal vaccine in the prevention of streptococcus disease in Nile tilapia, which could be used in different areas. This vaccine development could be effectively applied in the tilapia culture industry.

## 1. Introduction

Nile tilapia (*Oreochromis niloticus* Linn.) is cultured worldwide, mainly in China, Egypt, Vietnam, Bangladesh, Philippines, Indonesia, and Thailand. Global tilapia production in 2018 reached 4.5 million metric tons, corresponding to an estimated value of USD 6.0 billion, and the production has been definitely on an increasing trend [1]. In Thailand, the cultivation of tilapia has been increased rapidly and mostly practiced in cages and ponds by the intensive culture system. Fish in this culture system are prone to infections due to stress from many factors, i.e., high fish stock density and poor water quality, which also leads to rapid disease transmission by body contact. Therefore, disease control approaches are proven as an effective prevention method for most pathogenic diseases and have become achievable for fish health management in aquaculture all around the world.

Fish vaccines have proven to be effective means of preventing many major bacterial diseases in the aquaculture industry, such as vibriosis (*Vibrio anguillarum*) for the salmonid and cold-water marine fish species [2]. In the channel catfish industry, *Edwardsiella ictaluri* and *Flavobacterium columnare* are two of the most important pathogens [3,4] that vaccines have been developed for. Vaccine for streptococcosis has also been investigated for more than two decades since this disease has caused severe losses in many freshwater and marine aquaculture species [5,6,7,8,9,10].

Streptococcosis is considered a disease that can cause severe mortality among freshwater, estuarine, and marine fish species [11,12]. This disease is recognized as one of the major bacterial diseases in world tilapia cultivation and is caused by two major etiological agents, *Streptococcus agalactiae* and *Streptococcus iniae,* that are associated with septicemia and meningoencephalitis in fish [12]. The infected fish showed clinical signs of uni-or bilateral exophthalmia, haemorrhage, ascites, depression or excitability, anorexia, erratic swimming, and whirling, which can be observed soon after infection [13,14,15,16]. The outbreaks of streptococcosis have been related to environmental factors, including warm water temperatures, increased ammonia levels, and low dissolved oxygen [11]. *S. agalactiae* infections in tilapia have been reported in global aquaculture in different continents, especially Asia, including Thailand [17,18,19,20,21,22], Malaysia [23,24,25,26,27], Indonesia [28,29], Philippines [30], and China [31,32,33,34]. Importantly, the emergence of streptococcosis disease can take place anytime during tilapia farming, which may lead to the risk of disease distribution and loss of tilapia production if disease prevention and control are not well managed. Therefore, understanding tilapia’s response to the causative agents of streptococcosis together with a rationale vaccine strategy will provide key information on pathogenicity and immunogenicity, which will undoubtedly facilitate an effective vaccine design.

Currently, serotype Ia and serotype III are two major *S. agalactiae* serotypes associated with streptococcosis outbreaks in tilapia culture in Thailand [21]. However, there is a low number of reports on the comparative immunogenicity of both serotypes, which should lead to a better understanding of tilapia’s immune response against this pathogen. In this report, the protection of tilapia after being immunized by inactivated whole-cell *S. agalactiae* as a monovalent and bivalent vaccine against a particular pathogen was demonstrated. This information could facilitate the rationale for vaccine design which can be effectively applied in tilapia farming systems.

## 2. Materials and Methods

### 2.1. S. agalactiae and Experimental Fish

*S. agalactiae* serotype Ia and serotype III were isolated from diseased Nile tilapia (*O. niloticus* Linn.) during a streptococcosis outbreak in Thailand [21]. Experimental fish were provided by Kasetsart University, Kamphaeng Saen Campus, Nakhon Pathom Province. Juvenile tilapia used in all experiments had an average weight of 101.3 ± 3.7 g. Fish were randomly screened for *S. agalactiae* and other bacterial infections by bacteriological isolation from the liver, posterior kidney, and brain prior to the start of the experiment. Fish were acclimatized in cement tanks for two weeks before starting the tests. During the acclimatization, the fish were fed daily at 5% of commercial feed, and fish health was closely monitored. Then, 60 fish were placed separately in 12 cement tanks holding 800 L of water for 10 days before the beginning of the experiment and fed with commercial pellet feed twice a day.

### 2.2. Inactivated Whole-Cell S. agalactiae Preparation

A single colony of *S. agalactiae* serotype Ia and III cultured on brain heart infusion Agar (BHIA, Difco) was inoculated into 20 mL of brain heart infusion broth (BHIB, Difco). The starter culture was incubated in a shaking incubator at 32 °C for 18 h until the bacteria reached the mid-log phase. To prepare formalin-killed bacteria, 1.0 mL of starter bacterial suspension was inoculated into 300 mL of BHIB and continuously cultured in a shaking incubator at 32 °C for 18 h. Bacterial cells were collected by centrifugation at 1800× *g*, at 25 °C for 15 min, and a bacterial cell pellet was collected. Then, the bacterial cell pellet was washed and resuspended twice with 0.85% NaCl. The *S. agalactiae* cell suspension was treated with 1% formalin solution (within 0.85% NaCl) and kept at 4 °C overnight. Inactivated cells were tested for cell viability by spreading 0.1 mL formalin-killed bacteria on BHIA and incubating at 37 °C for 24 h. The success of cell inactivation was indicated by the lack of growth on BHIB. After completion of cell inactivation, excessive formalin was removed by washing with 0.85% NaCl twice. For monovalent vaccine preparation, the bacterial cells were adjusted to an absorbance of 0.67 at 600 nm to obtain an approximate cell number of 1 × 10^9^ CFU mL^−1^. For bivalent vaccine preparation, both *S. agalactiae* serotypes Ia and III were combined, and the absorbance was measured at 600 nm to obtain an approximate cell number of 1 × 10^9^ CFU mL^−1^.

### 2.3. Evaluation of Vaccine Efficacy in Laboratory Scale: S. agalactiae Challenge Test

#### 2.3.1. Immunization with Monovalent and Bivalent Streptococcal Vaccine

To determine the response of tilapia against inactivated *S. agalactiae* vaccine, the fish were immunized with a monovalent vaccine and bivalent vaccine. Briefly, a total of 720 juvenile tilapia were separated into four groups (60 fish/tank, with 3 replicates), including 0.85% NaCl (control); monovalent vaccine: serotype Ia (T1); monovalent vaccine: serotype III (T2); and bivalent vaccine (T3). For immunization, fish were anesthetized with 200 mg L^−1^ Aqui-S (Aqui-S New Zealand LTD, Lower Hutt, New Zealand) by immersion method. Then, fish were intraperitoneally injected (IP) with 0.2 mL of monovalent or bivalent vaccine or 0.85% NaCl (control). All fish were kept in cement tanks, supplied with a flow-through water system and continuous aeration. Fish were fed with a 5% feeding rate of a commercial pellet feed twice a day. The water temperature in experimental tanks was maintained by a heater at 30 ± 2 °C.

#### 2.3.2. Challenge Test

After 30 and 60 days of immunization, 60 fish from each experimental group were divided into two groups (30 fish/group, 10 fish/fiberglass tank holding 200 L water, with 3 replicates) and challenged with serotypes Ia and III. The fish were anesthetized by immersion in 200 mg L^−1^ Aqui-S (Aqui-S New Zealand LTD, Lower Hutt, New Zealand). The bacterial challenge was conducted by intraperitoneal injection (IP) with 0.2 mL of *S. agalactiae* inoculum at 1 × 10^8^ CFU mL^−1^ (equivalent to 2 × 10^7^ CFU per fish). Mortalities and survival rates were recorded over a period of 14 days after the challenge. The temperature during the challenge period was maintained at 30 ± 2 °C. The mean percentage of cumulative mortality was compared between each vaccine and control group. The dead fish were collected to confirm the cause of infection by the bacterial re-isolation from posterior kidney, spleen, liver, and brain specimens on BHI agar. The efficacy of the vaccine was calculated as relative percent survival (RPS) as described by Amend (1981) [35] using the following formula:

RPS = {1 − (% mortality of vaccinated fish ÷ % mortality of control fish)} × 100(1)

### 2.4. Determination of Serum Antibody Titer

Serum antibody titer was determined every week after immunization for 8 weeks. Serum from 10 fish of each experimental group and control were individually tested with both serotypes of *S. agalactiae* by agglutination test. Briefly, the blood of tilapia was withdrawn from the caudal vein with a 1.0 mL syringe, transferred to a 1.5 mL microcentrifuge tube, and left for clotting at room temperature for 60 min. Then, the samples were centrifuged at 600× *g* at 25 °C for 15 min to collect the serum.

Antibody titer was determined with the agglutination method using a 96-well round bottom plate with serial two-fold dilution. A total of 50 μL of immunized tilapia serum were added to the 1st and 2nd wells of each row, and 50 μL of 0.85% sodium chloride was added to wells 2–12. Sera were continually diluted in multi-wells by two-fold dilution using 0.85% sodium chloride. For this antibody titer analysis, the tilapia sera of each group were divided into 2 subgroups for testing with 2 different antigens (inactivated *S. agalactiae* serotype Ia and serotype III). Then, 50 μL of inactivated 1 × 10^8^ CFU/mL bacterial cell suspension was added to each well and mixed. The plate was covered and incubated overnight at 32 °C. Agglutination antibody titer was reported as the highest dilution of the serum that showed agglutination of antigen. Serum + 0.85% sodium chloride was used as positive control, and antigen + 0.85% sodium chloride was set as negative control.

### 2.5. Evaluation of Vaccine Efficacy in Farm Scale

**Figure 1:**
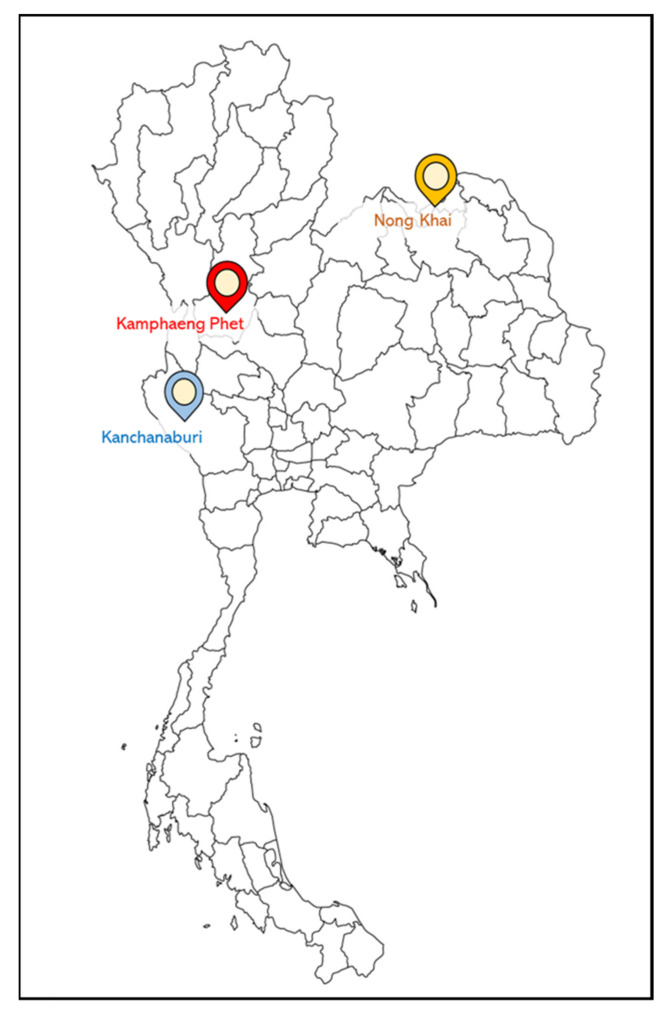
Three different locations of vaccine field trials in Thailand: north (Kamphaeng Phet province), northeast (Nong Khai province) and west (Kanchanaburi province).

**Figure 2:**
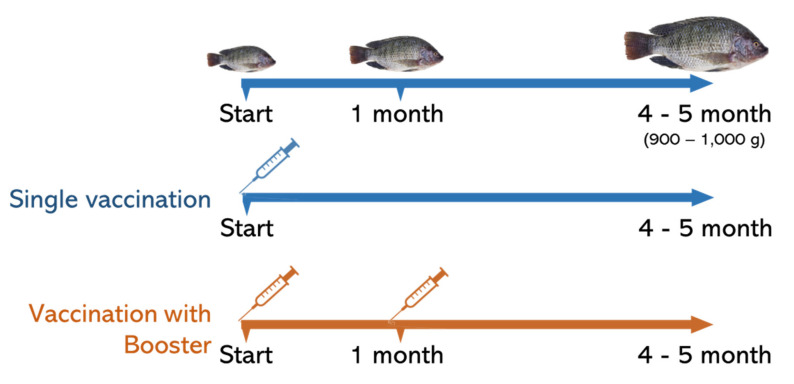
Schematic diagram of vaccination plan for evaluating bivalent streptococcal vaccine efficacy by single and booster vaccination.

The field trial for vaccine efficacy was conducted in three different parts of Thailand, including the northeastern part (Nong Khai province), northern part (Kamphaeng Phet province) and western part (Kanchanaburi province) (Figure 1). For the evaluation of vaccine efficacy in these provinces, the trial of the bivalent vaccine was evaluated by single and booster vaccination. Briefly, before vaccination, 5000 juvenile Nile tilapia with an initial weight of about 25–30 g were anesthetized by immersion in 200 mg L^−1^ Aqui-S (Aqui-S New Zealand LTD, Lower Hutt, New Zealand). Then, they were IP immunized with 0.2 mL bivalent vaccine. All fish were immediately transferred to floating cages (5 × 5 × 2.5 m) and separated as 2500 fish per cage. In the case of single vaccination, vaccinated fish were cultured until fish size reached 900–1000 g. In the case of booster vaccination, 1 month after the first vaccination, 2500 fish were IP immunized again with 0.2 mL bivalent vaccine and transferred back to the cage. Vaccinated fish were continuously cultured until fish size reached 900–1000 g. The evaluation was recorded as percent survival at the end of cultivation. These farm trials were conducted for two crops of cultivation in March and November (Figure 2). In Kamphaeng Phet, the temperature during the trial was 25.67–36.2 °C and 15.6–34.0 °C for the March and November crops, respectively; in Nong Khai, the temperatures were 24–33.4 °C and 16.7–33.0 °C, respectively, and in Kanchanaburi, the temperatures were 27.33–35.11 °C and 20.55–34.0 °C, respectively. The evaluation of the cause of fish death during the field trial was accomplished by observation based on the signs and symptoms of diseased fish. Occasionally, to verify the authentic species and serotype of the pathogen, the moribund fish were subjected to pathogen isolation and characterization by microbiology and molecular biology techniques.

### 2.6. Average Weight Gain of Vaccinated Fish

Juvenile tilapia, with an initial weight of 20 g, were IP immunized with 0.2 mL bivalent vaccine or non-vaccinated fish. All fish were immediately transferred to floating cages (5 × 5 × 2.5 m) and separated as 2500 fish per cage for 4 replicates. Vaccinated fish and non-vaccinated fish were cultured in Kanchanaburi province, Thailand, for 6 months from October 2021 to March 2022. At the end of the experimental period, fish were harvested, and all fish were weighed. Survival rates were compared.

### 2.7. Statistical Analysis

The mean ± standard deviation of each parameter was calculated for each treatment. Data were statistically compared by one-way ANOVA, and Duncan’s new multiple range test was used for multiple comparisons of the treatments [36] using SPSS 16.0 software (IBM Corporation, Chicago, IL, USA). The results were considered statistically significant with *p* < 0.05. The Shapiro–Wilk test was used to determine normal distribution.

## 3. Results

### 3.1. Specific Serum Antibody Titer of Tilapia Vaccinated with Monovalent and Bivalent Vaccine

**Figure 3:**
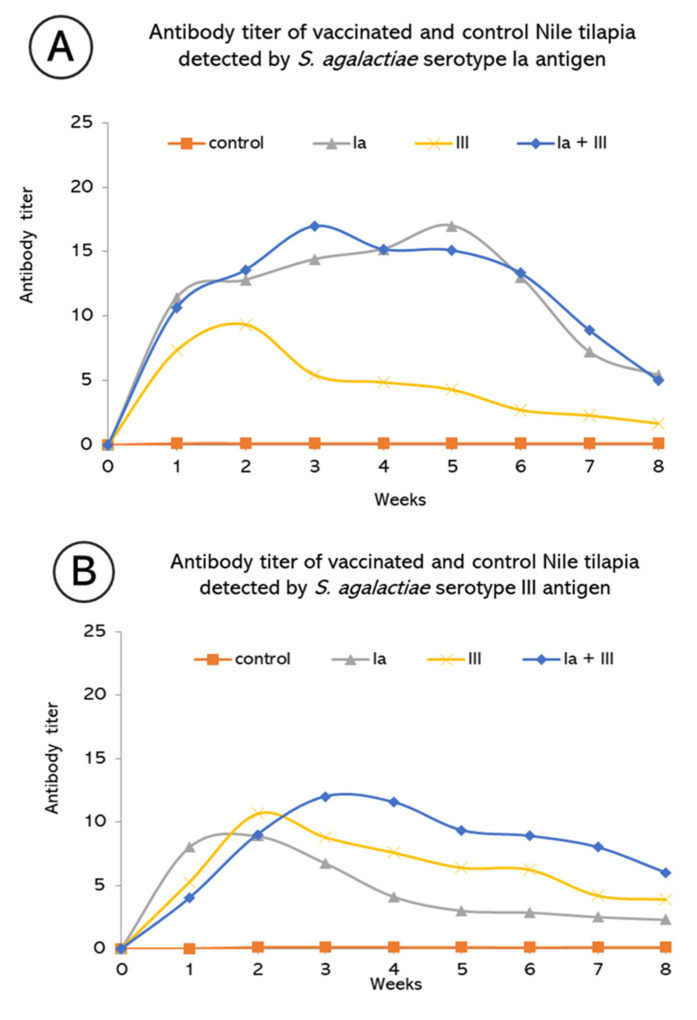
Antibody titer of Nile tilapia (*Oreochromis niloticus* Linn.) immunized with formalin-inactivated vaccine of *S. agalactiae* serotype Ia and III. Titer was detected with two different antigens (**A**) serotype Ia (**B**) serotype III. Data were reported as mean ± SD (*n* = 6).

Levels of antibody titer of all vaccinated groups were higher than in the control group, for which antibody serum titer could not be detected throughout the experimental period (*p* < 0.05). Maximum levels of specific antibodies against *S. agalactiae* serotypes Ia and III were detected at the 5th or 2nd week after vaccination with particular vaccines. Interestingly, cross-reactivity against different serotypes was observed but with a lower titer level than homologous antigens. The bivalent vaccine showed a remarkable increase in antibody titer compared with monovalent vaccines, especially against serotype III (Figure 3A,B).

### 3.2. Evaluation of Vaccine Efficacy: Laboratory Test

#### 3.2.1. Percent Cumulative Mortality

**Figure 4:**
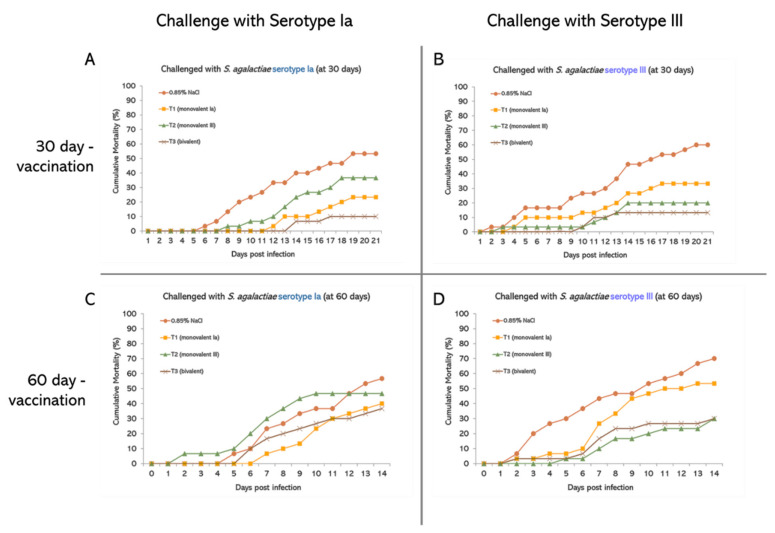
Mean cumulative mortality (%) of Nile tilapia (*Oreochromis niloticus* Linn.) immunized with formalin-inactivated vaccine and challenged with *S. agalactiae* serotypes Ia and III at 30 and 60 days post-vaccination. (**A**) Challenged with *S. agalactiae* serotype Ia at 30 days, (**B**) challenged with *S. agalactiae* serotype III at 30 days, (**C**) challenged with *S. agalactiae* serotype Ia at 60 days, and (**D**) challenged with *S. agalactiae* serotype III at 60 days (*n* = 30).

Percent cumulative mortality of non-vaccinated fish (0.85% NaCl) showed approximately 50% mortality in the serotype Ia- and 60–70% mortality in serotype III-challenged group. Serotype III infection could induce fish mortality from day 2 of the challenge, and mortality could be continuously observed thereafter. Similarities were observed in the serotype Ia challenge, except the mortality was delayed, starting on days 5–6 after the challenge. This result affirmed the greater virulence of serotype III than serotype Ia (Figure 4A–D).

At the end of the 14-day challenge, the percent cumulative mortality of all vaccinated groups (T1, T2, and T3) showed a reduction in fish mortality compared to the non-vaccinated group (0.85% NaCl) both 30 and 60 days post-vaccination. With the exception of the T2 vaccinated group at 60 days, higher percent cumulative mortality than the non-vaccinated was observed, in which mortality started from days 2 post-challenge (Figure 4C). However, T1 and T2 could decrease fish mortality after homologous serotype challenge compared to the non-vaccinated group. T3 showed the lowest fish mortality from *S. agalactiae* challenge, especially at 30 days but not at 60 days (Figure 4A–D). The vaccine appeared to be more effective at 30 days than 60 days post-vaccination. The protection against *S. agalactiae* was lower when the days after vaccination were longer. Interestingly, the efficacy appeared to be positively related to the antibody titer after vaccination.

#### 3.2.2. Percent Survival

**Figure 5:**
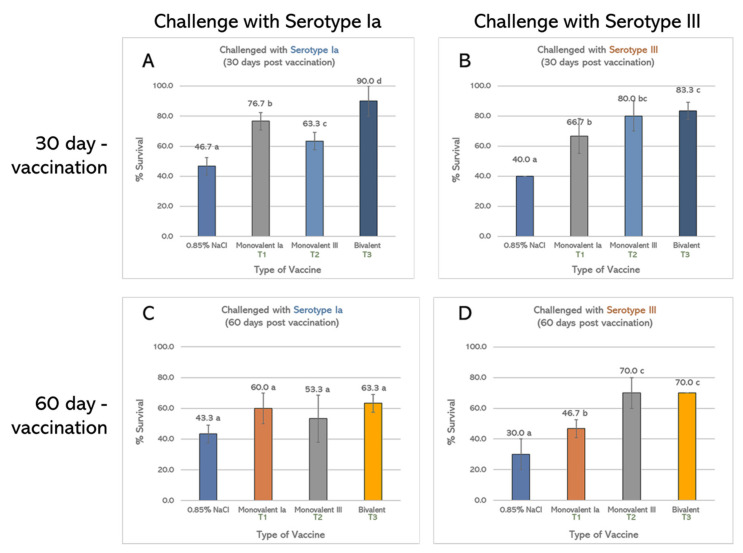
Percent survival of Nile tilapia (*Oreochromis niloticus* Linn.) immunized with monovalent vaccine: serotype Ia (T1); monovalent vaccine: serotype III (T2), bivalent vaccine (T3), and non-vaccinated (control). After vaccination at 30 days, fish were challenged with *S. agalactiae* serotype Ia (**A**) and serotype III (**B**). After vaccination for 60 days, fish were challenged with *S. agalactiae* serotype Ia (**C**) and serotype III (**D**) (*n* = 30). a, b, c, and d = significant difference (*p* < 0.05).

At 30 days post-vaccination, all vaccinated groups exhibited a significantly higher survival rate (*p* < 0.05) than the non-vaccinated group after being challenged with two serotypes of *S. agalactiae*. T1 showed 76.7% survival after the serotype Ia challenge, whereas T2 showed 80.0% survival after the serotype III challenge. However, T1 demonstrated 66.7% survival after the serotype III challenge, and T2 demonstrated 63.3% survival after the serotype Ia challenge. The significantly highest survival rate was observed in T3-vaccinated fish after the challenge with serotype Ia (90.0%) and serotype III (83.3%) (Figure 5A,B). At 60 days after vaccination, the percent survival rates of all vaccinated groups after the challenge with serotype Ia were not significantly different from the non-vaccinated group (*p* > 0.05). However, significant differences were still found with the serotype III challenge (70% survival) (*p* < 0.05) (Figure 5C,D). Moreover, the overall percent survival of vaccinated groups was reduced at 60 days post-vaccination.

#### 3.2.3. Relative Percent Survival (RPS)

**Figure 6:**
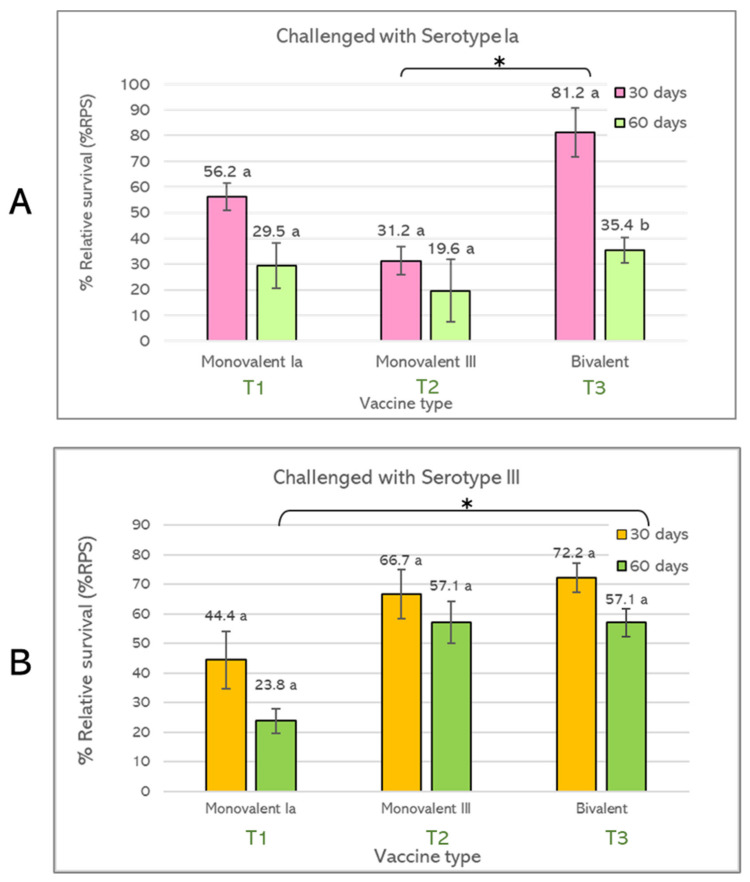
Relative percent survival (RPS) of Nile tilapia (*Oreochromis niloticus* Linn.) immunized with monovalent vaccine: serotype Ia (T1); monovalent vaccine: serotype III (T2) and bivalent vaccine (T3), and non-vaccinated (control). After a single shot of vaccination at 30 and 60 days, fish were challenged with *S. agalactiae* serotypes Ia and III. (**A**) RPS of Nile tilapia challenged with *S. agalactiae* serotype Ia, (**B**) RPS of Nile tilapia challenged with *S. agalactiae* serotype III. (*n* = 30). a, b = significant difference between 30 and 60—days (*p* < 0.05) and asterisk (*) = significant difference between treatment groups (*p* < 0.05).

Relative percent survival (% RPS) of T1, T2 and T3 after challenge with *S. agalactiae* serotype Ia and serotype III at 30 and 60 days post-vaccination was evaluated. The computed results showed no significant differences amongst vaccinated groups. However, only the T3-vaccinated group showed significant differences in % RPS between 30 (81.2% RPS) and 60 days (35.4% RPS) post-vaccination after the challenge by serotype Ia (Figure 6A,B).

Comparing % RPS among different vaccine types, it is shown that T3 (bivalent vaccine) could increase % RPS greater than T1 and T2. A significant increase in % RPS was observed in bivalent vaccine after the challenge with serotype Ia (81.2% RPS at day 30 post-vaccination) compared with T2 (monovalent III) (31.2% RPS) and after challenge with serotype III (57.1% RPS at day 60 post-vaccination) compared with T1 (monovalent Ia) (23.8% RPS), respectively (Figure 6A,B).

### 3.3. Evaluation of Vaccine Efficacy: Farm Scale

Bivalent streptococcal vaccine efficacy was evaluated in Nile tilapia cultivated on local farms in 3 different locations in Thailand. Two programs of vaccination, a single vaccination and a vaccination with a booster, were set up to demonstrate the different effects of the vaccine in altering production yield (fish size reached 900–1000 g).

**Figure 7:**
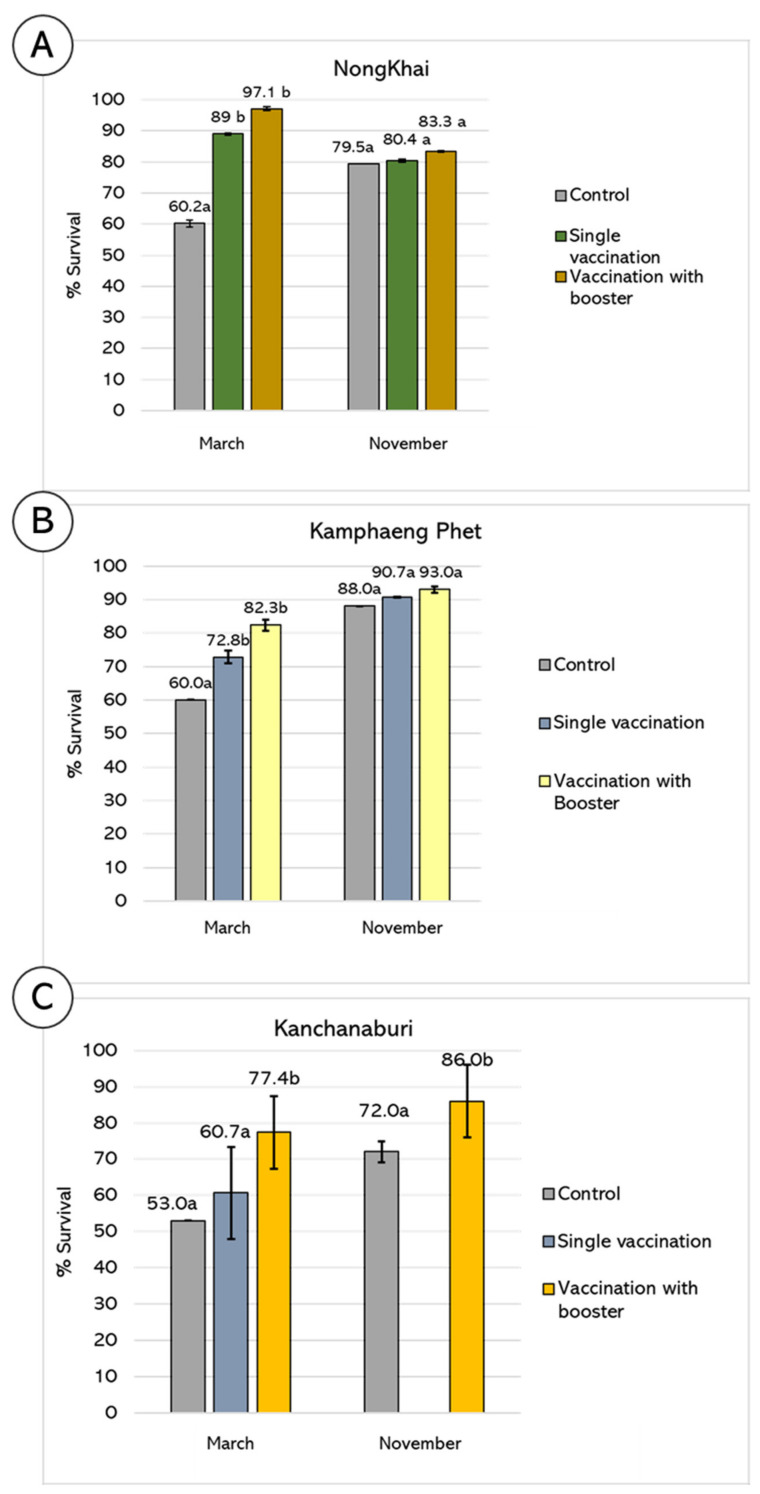
Field trial of bivalent streptococcal vaccine in 3 different locations in Thailand. Percent survival of Nile tilapia after single vaccination or vaccination with booster was evaluated in March and November 2015. (**A**) Survival rates of vaccinated tilapia in Nong Khai province. (**B**) Survival rates of vaccinated tilapia in Kamphaeng Phet province. (**C**) Survival rates of vaccinated tilapia in Kanchanaburi province. a, b = significant difference between treatment group in each month (*p* < 0.05).

Vaccinated and non-vaccinated tilapia cultivated in Nong Khai province, northeastern Thailand, were cultured in floating cages in the Khong river. In the March crop, the vaccinated groups clearly showed a significant increase in survival rate over the non-vaccinated group at 97.1, 89.0 and 60.2%, respectively (*p* < 0.05), while no significant differences were found between the single and vaccination with booster groups (Figure 7A). However, in the November crop, survival rates of vaccinated and non-vaccinated groups were not significantly different (*p* > 0.05).

In Kamphaeng Phet province, vaccinated fish were cultured in floating cages in the Ping River. The results were quite similar to Nong Khai province, in which significant differences in survival rates were found between vaccinated and non-vaccinated groups only in March at 82.3, 72.8 and 60.0% for the vaccination with a booster, single vaccination and non-vaccinated groups, respectively (*p* < 0.05) but not in November at 93.0, 90.7 and 88.0%, respectively (*p* > 0.05) (Figure 7B). Again, the percent survival of vaccinated fish was not significantly different between the single vaccination group and the vaccination with a booster group in both March and November. Meanwhile, the overall production yield of tilapia in November was higher than in March (Figure 7B).

In Kanchanaburi province, the results were quite different; significant differences in survival rates were found in both March and November crops (Figure 7C). In the March crop, the survival rates of the vaccination with a booster, single vaccination and non-vaccinated groups were 77.4, 60.7 and 53.0%, respectively (*p* < 0.05). Furthermore, the group receiving a vaccination with a booster had a significantly higher survival rate than the single vaccination group (*p* < 0.05). Since in this crop, the survival rate of the single vaccination group showed no significant difference from the non-vaccinated group, we decided to test the efficacy of only vaccination with a booster in November, which showed a percent survival of 86%; this was significantly higher than the non-vaccinated group (72.0%) (*p* < 0.05) (Figure 7C).

Most of the moribund and dead fish showed clinical symptoms of streptococcosis, including spiral swimming, a darkening body and some with ascitic and exophthalmic conditions (data not shown). Some of the moribund fish were subjected to isolating pathogens based on microbiology and molecular techniques. It was demonstrated that most of the infections were caused by *S. agalactiae* of both serotype Ia and III, while minor cases were caused by *Flavobacterium columnare* and Tilapia Lake virus with no incidence of co-infection. The incidences of these three diseases were 87.5%, 9.25% and 3.25%, respectively.

### 3.4. Average Weight Gain of Vaccinated Fish

**Figure 8:**
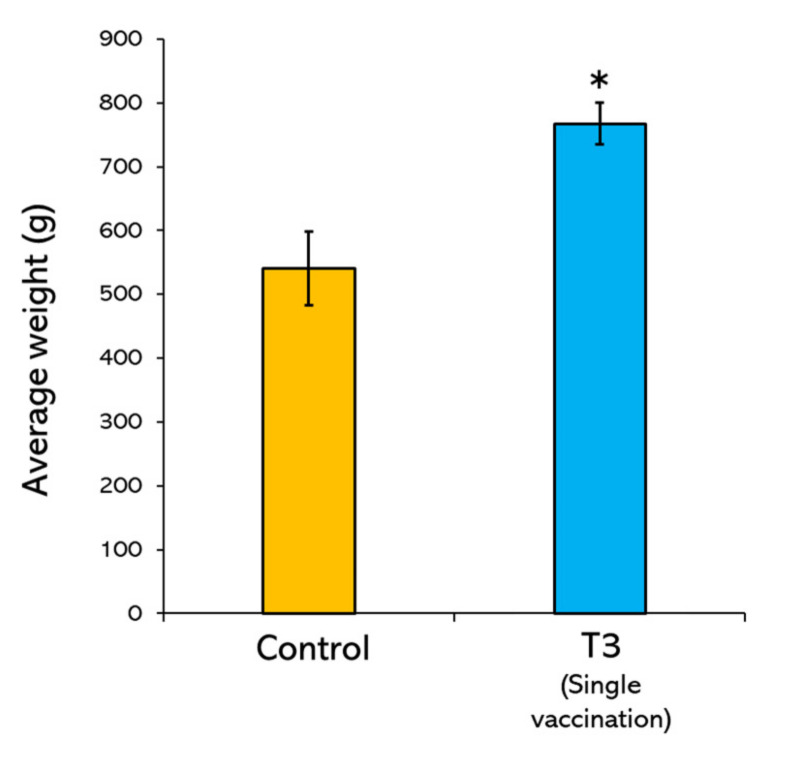
Average weight of Nile tilapia single vaccinated with bivalent vaccine and non-vaccinated (control) in field trial. The fish were cultivated for 6 months in Kanchanaburi province Thailand during October 2021–March 2022. Asterisk (*) indicated the significant difference between vaccinated and non-vaccinated fish (*p* < 0.05).

For the trial in Kanchanaburi province, after 6 months of cultivation, the average weight of non-vaccinated (control) and bivalent vaccine injected fish were compared. Interestingly, the vaccinated fish were significantly higher in their average weight (767.25 ± 16.36 g) than the control group (540.65 ± 28.93 g) (*p* < 0.05) (Figure 8). This result demonstrated that the bivalent vaccine injection could provide a positive effect on the fish size.

## 4. Discussion

*S. agalactiae* is a pathogenic bacterium that causes streptococcosis disease, which is generally associated with massive mortalities in the aquaculture industry of Southeast Asia and other continents. This disease outbreak commonly affects tilapia cultivation, causing massive economic losses. Streptococcosis is one of the major bacterial diseases that can retard tilapia production in most regions of the world. To combat this disease, a practical vaccine should be developed to provide promising streptococcal preventive and control measures in tilapia culture.

Based on our research on *S. agalactiae* characterization, we could isolate 2 major serotypes, serotype Ia and serotype III, that caused disease in tilapia, which was similar to the surveillance in other countries in SE Asia such as Malaysia [17,21,22,37,38,39]. Based on our findings, both serotypes showed similar distribution throughout Thailand during 2012–2014; however, it was found that serotype III exhibited higher virulence than serotype Ia [21]. To develop a streptococcal vaccine that could be used throughout Thailand and other countries, an inactivated vaccine was designed based on the distribution pattern of *S. agalactiae* and understanding the basis of the fish immune response against both serotypes.

The development of the *S. agalactiae* vaccine has been investigated for decades since the demand for tilapia has been increasing drastically worldwide. This has led to the growing trend of tilapia culture in many parts of the world. *S. agalactiae* vaccine development for Nile tilapia has progressed rapidly in association with the new technologies that have been adopted, as reviewed by many reports [40,41,42,43,44]. However, most of the developed vaccines were only tested on a laboratory scale, and there was only a feed-based streptococcal vaccination in tilapia farms reported in Malaysia [45].

To design the vaccine that could be used in several cultivation regions, the essential antigenic component of the inactivated vaccine should be optimized (1) to induce an appropriate immune response and (2) to provide promising protection. Recently, inactivation methods of the *S. agalactiae* vaccine were compared between formalin, hydrogen peroxide, and pH manipulation, which showed similar efficacy of protection in Nile tilapia [46]. Besides the inactivated vaccine, there are other types of *S. agalactiae* vaccine for Nile tilapia generated from live-attenuated [47,48], recombinant and DNA vaccines [49,50,51]. In this study, monovalent (serotype Ia or serotype III) and bivalent vaccines (combined serotype) were prepared by a formalin-inactivated protocol, and both of them were tested for their ability to induce a specific immune response (immunogenicity) against serotype Ia or serotype III.

Upon immunization with monovalent and bivalent vaccines, specific antibodies in all immunized groups were increased with different patterns compared with the control group. Interestingly, fish immunization with a bivalent vaccine showed a greater level of antibody titer with serotype III along 8 weeks of determination. This observation showed the discrepancy in tilapia immune response against serotype Ia and serotype III. It might be explained by the common antigenic molecules found in both serotypes. However, the number of antigens in serotype Ia might be more dominant than antigens from serotype III, which could efficiently activate specific immunity [49]. Therefore, it would be speculated that, regarding overall specific antibodies, the majority arose from serotype Ia; hence, a similar level of antibody titer was observed after reacting with serotype Ia but not with serotype III. This observation showed the characteristic of tilapia, which resembles most teleosts, which are considered to be low responders with consideration to antibody production.

Regarding the protection of monovalent and bivalent vaccines, it was clearly shown that the bivalent vaccine showed superior efficacy against both serotypes. This cross-protection resembled the cross immunoreactivity to different serotypes of *S. agalactiae*. It would further be affirmed that both reactions resulted from the sharing of common antigenic molecules between both serotypes [21,49]. Hence, it would be concluded that antibody titer is a primary and direct immune index that could be used to indicate the specific response to invading organisms and used as a guideline for vaccine formulation. Moreover, it is quite interesting to note that most of these vaccines studied on a laboratory scale showed good potency in Nile tilapia with significant antibody response and RPS. It is a fact that excellent protection has resulted from the same serotype of vaccines and emerging bacteria. On the other hand, it should also be noted that this is the first report comparing monovalent and bivalent streptococcal vaccines based on vaccine efficacy and immunoreactivity. Therefore, upon this observation, the bivalent vaccine was further evaluated for its efficaciousness on tilapia farms.

For the effective application in the cultivation system, the efficacy of the vaccine would depend on the occurrence of disease emergence, in which different serotypes of *S. agalactiae* would occasionally be varied. The trial was planned to surpass the period of high risk of streptococcal disease (March) and low risk of disease emergence (November). Interestingly, our bivalent vaccine exhibited percent survival ranging from 60.7 to 90.7% survival by single vaccination and 77.4 to 97.1% survival by vaccination with a booster. Meanwhile, the unvaccinated group showed only 53.0–88.0% survival. These differences varied according to the month of crop and experimental sites. The season might be the most influential factor that could impact the vaccine efficacy in this trial. *S. agalactiae* in Thailand has been reported to cause serious damage during the summer (29, 42). From our results, the bivalent vaccine showed high efficacy during the March crop but not in the November crop. In the March crop, the average temperature of three locations was 30.3 °C, while the average temperature of the November crop was 25.6 °C. Additionally, it is interesting to note that in the November crop, the efficacy of our bivalent vaccine appeared to be not significant when compared with the non-vaccinated group, especially in Nong Khai and Kamphaeng Phet provinces. This might be explained by the lower temperature during that period, which lessened the virulence of *S. agalactiae*. However, the November crop in Kanchanaburi province still showed significant efficacy of the vaccine with a booster over the non-vaccinated group. This might be due to the fact that many large-sized farms were located within this testing site, which could severely impact culture conditions, such as water quality and water flowing systems that could facilitate disease transmission within the area.

Tilapia vaccine efficacy at the farm level has been tested in farms endemic for streptococcosis in Malaysia during April and July. It would be suggested that our injected vaccine has similar efficacy to the oral streptococcal vaccine, either with a single booster or double booster [45]. As compared with the oral vaccine, our bivalent vaccine, which was formulated with the most prevalent and virulent serotypes, would enhance the vaccine efficacy and promptly provide protection to any occasionally emerging disease. Moreover, our bivalent vaccine did not apply adjuvants, which would lower the cost of the vaccine and the application of the vaccine, resulting in a larger size of vaccinated fish. It is affirmed that our vaccine formula could protect from streptococcosis disease caused by heterologous serotypes in different locations, increase the production yield, and increase the average weight of vaccinated tilapia in the field trial. However, it should be mentioned that the injection vaccine is not easy to conduct when compared with oral vaccination, but it would be appropriately used as primary vaccination prior to boosting by other routes of vaccination during cultivation or used in a specific stage of fish production, such as in brooder.

Taken together, the results in this study demonstrated the basic knowledge of comparative immunity in tilapia immunized with a monovalent vaccine (serotype Ia and III), which showed a limitation of vaccine efficacy produced from a single serotype. Moreover, our findings clearly indicated the superiority of the bivalent vaccine in the protection of both serotypes after a single immunization. In the present study, we used a formalin-inactivated whole-cell vaccine method that is conventional and inexpensive. The efficacy of the formalin-killed vaccine was well correlated with its ability to stimulate the humoral response by producing specific antibodies. The efficacy of the bivalent vaccine against serotypes Ia and III found in this study should be a stepping stone for the rational, strategic plan of a vaccination program for the tilapia culture industry, especially in countries with the existence of these two serotypes. The vaccine efficacy was investigated at the farm level, which showed protection throughout the crop. However, an adjuvant application may be the next target that may enhance the protection provided by a single immunization. A booster vaccination is another rational approach, but it should be practical and effective with the culturing system, such as via oral administration.

## Data Availability

The authors declare that the data supporting the findings of this study are available within the figures and tables. All data are available from the corresponding author upon reasonable request.
